# Correction: What works in engaging communities? Prioritising nutrition interventions in Burkina Faso, Ghana and South Africa

**DOI:** 10.1371/journal.pone.0300859

**Published:** 2024-03-14

**Authors:** The PLOS ONE Staff

## Notice of Republication

This article was republished on March 4, 2024, to correct an error in the author list. Aviva Tugendhaft, on behalf of the INPreP study group, was erroneously not included as the last author. The publisher apologizes for this error. Please download this article again to view the correct version. Both the originally published uncorrected article and the republished corrected article are provided here for reference.

## Supporting information

S1 File Originally published, uncorrected article. (PDF)

S2 File Republished, corrected article. (PDF)
