# Supplementary figures and images for: What works in engaging communities? Prioritising nutrition interventions in Burkina Faso, Ghana and South Africa
Source: PLoS One. 2023 Dec 13;18(12):e0294410. doi: 10.1371/journal.pone.0294410 (PMC10718458; doi:10.1371/journal.pone.0294410)

**CHAT participant manual Nanoro, Burkina Faso**


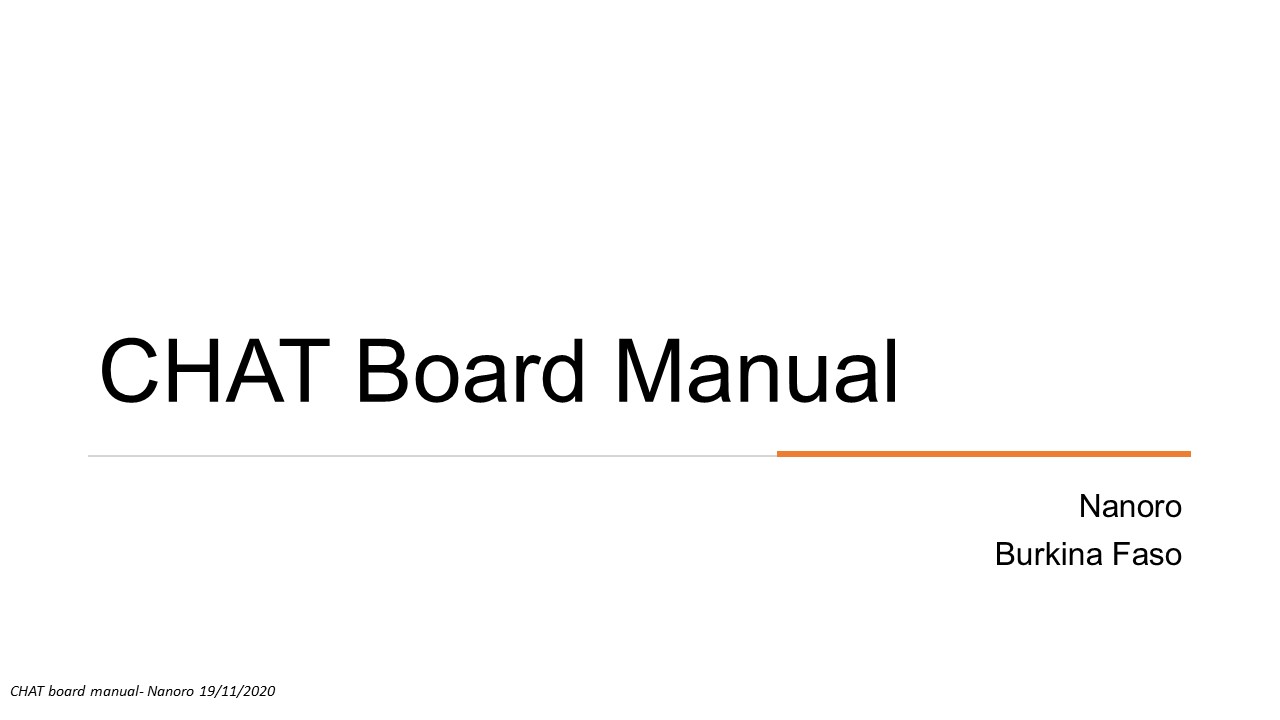


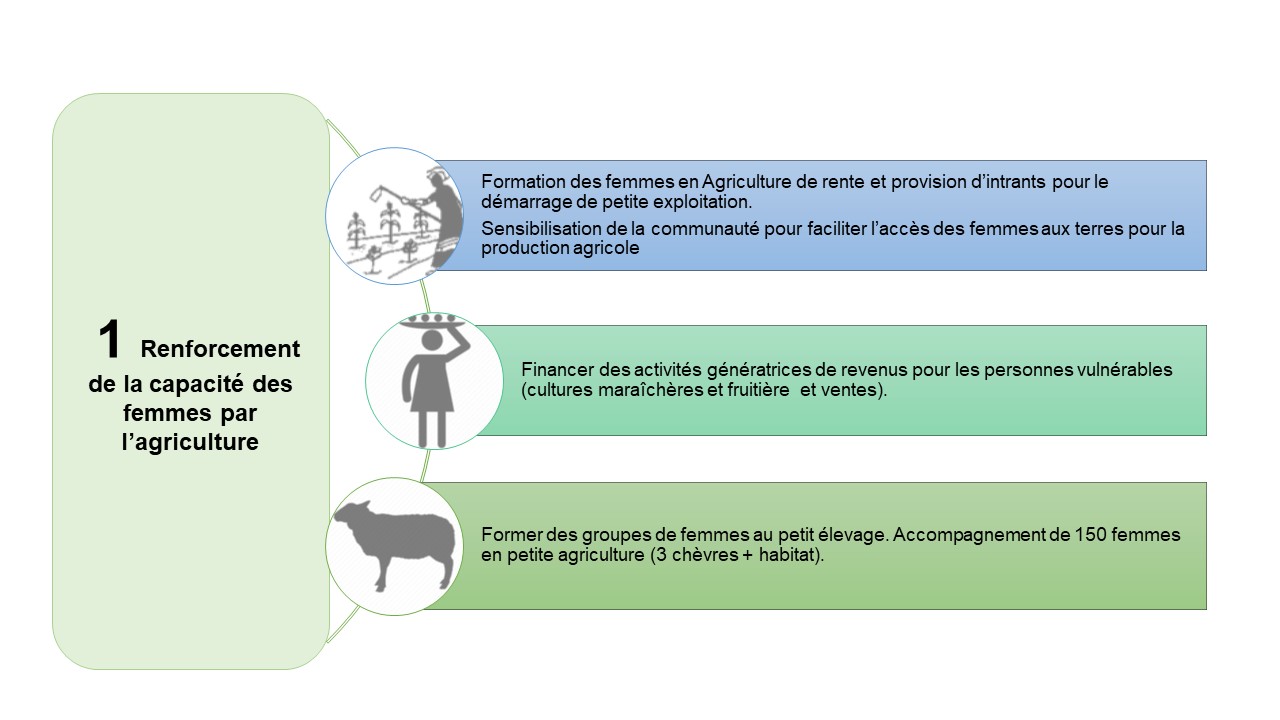


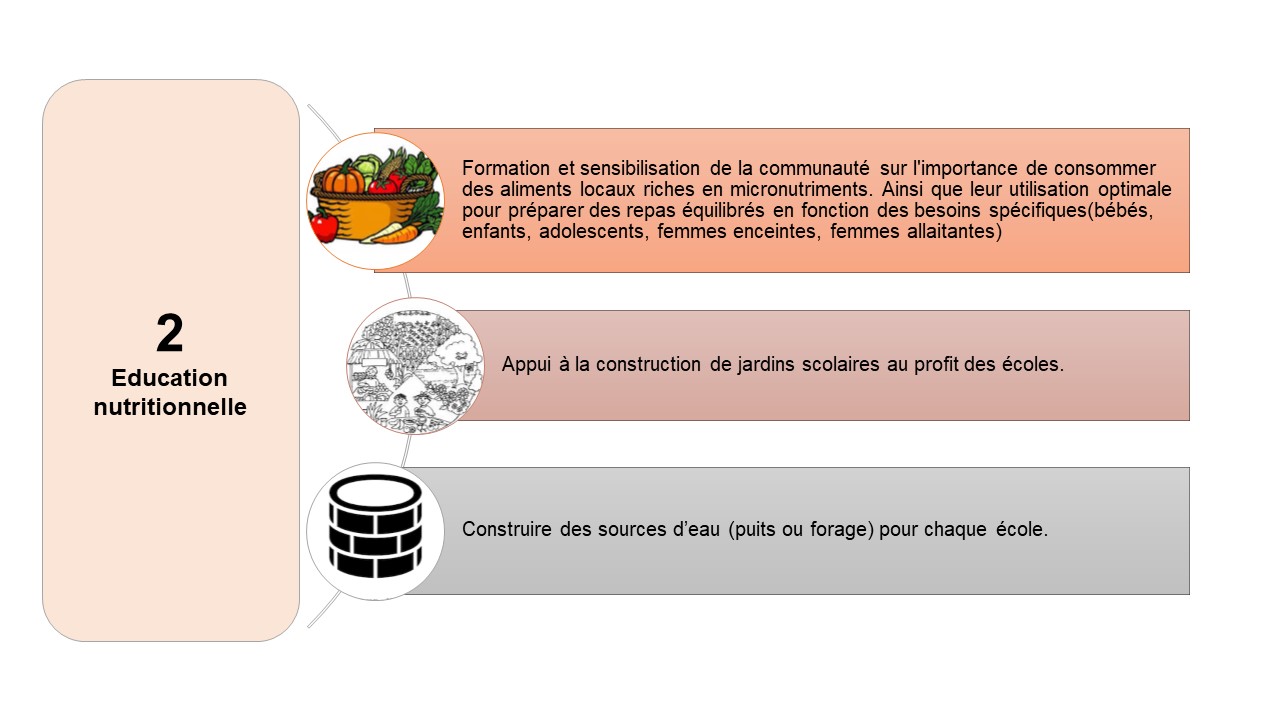


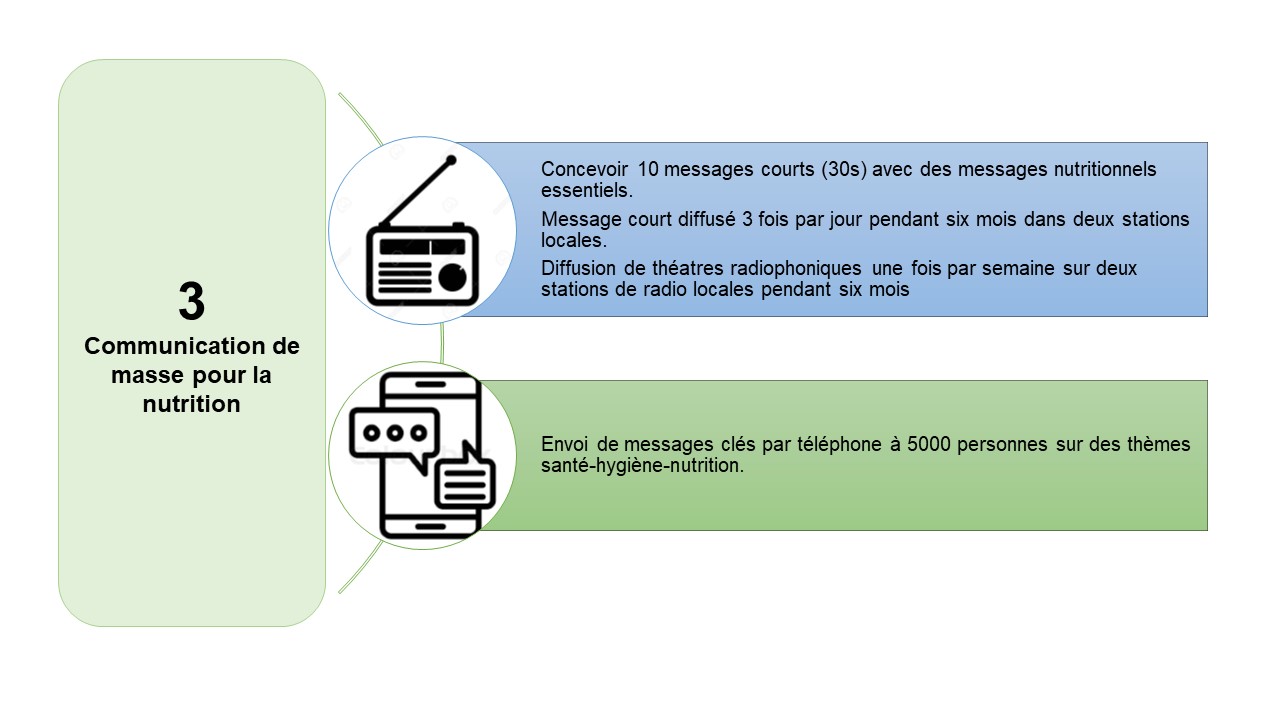


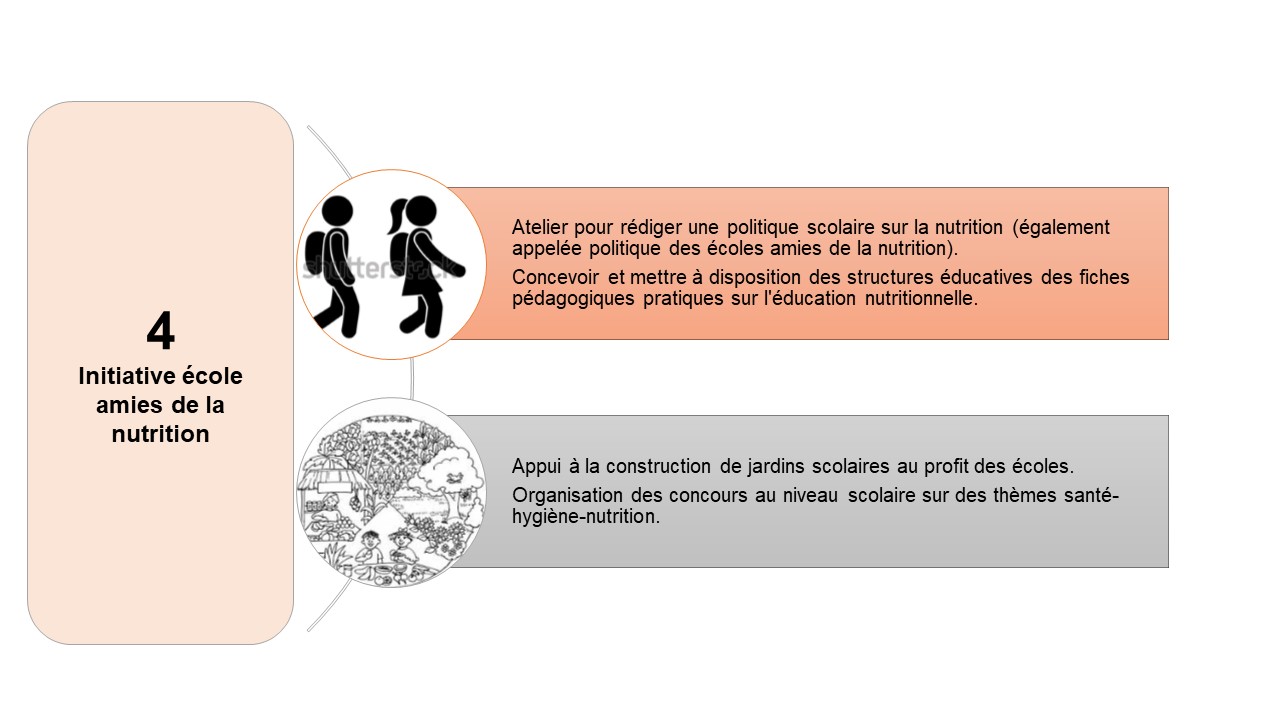


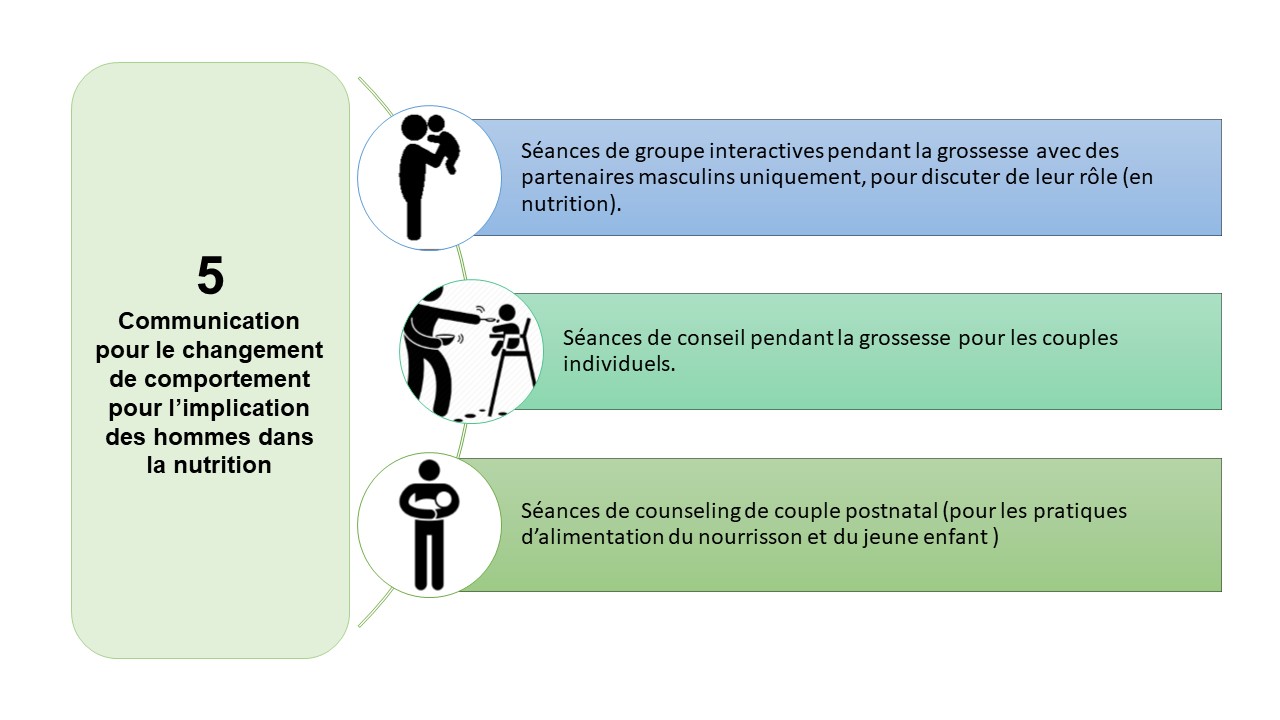


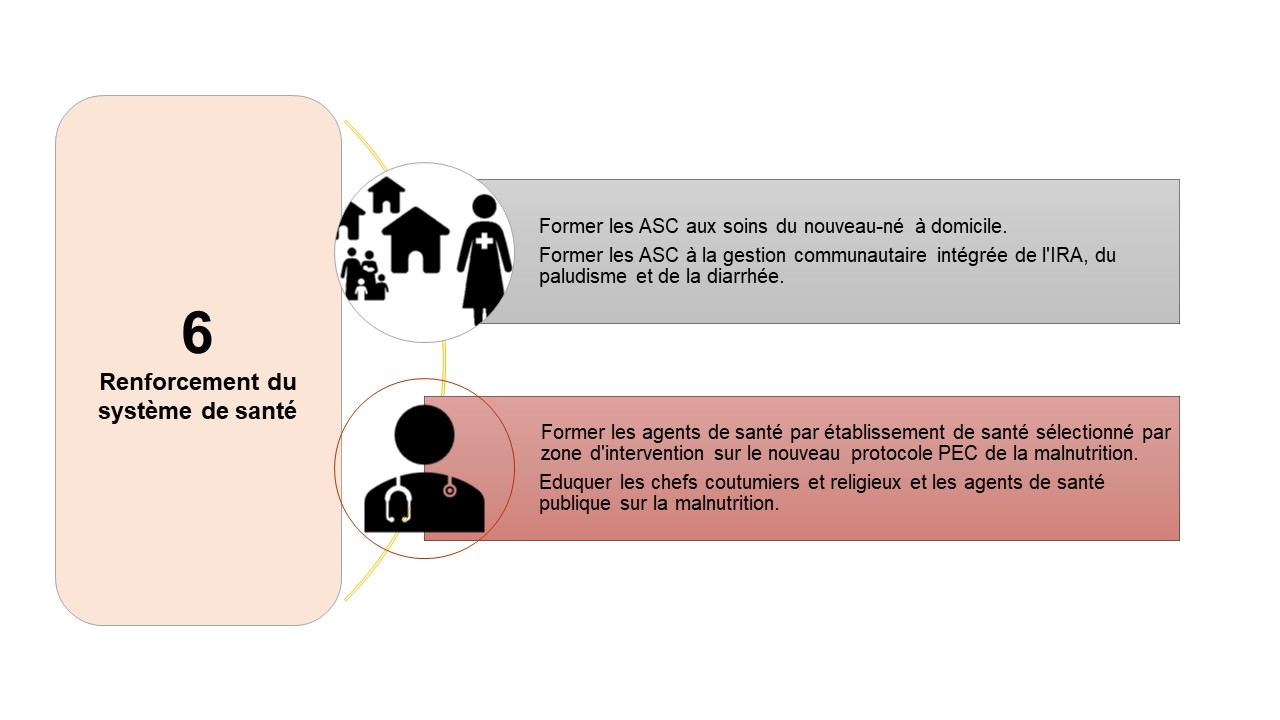

Supplement: S1 Appendix — (DOCX) [file pone.0294410.s001.docx]
