# Supplementary material for: What works in engaging communities? Prioritising nutrition interventions in Burkina Faso, Ghana and South Africa
Source: PLoS One. 2023 Dec 13;18(12):e0294410. doi: 10.1371/journal.pone.0294410 (PMC10718458; doi:10.1371/journal.pone.0294410)
Supplement: S2 Appendix — (DOCX) [file pone.0294410.s002.docx]

**Appendix B CHAT participant manual Navrongo, Ghana**

|  | **Words** | **Sticker value (N=104)** | **Narrative** | **Cost** |
| --- | --- | --- | --- | --- |
| 1 | Community nutrition education |  |  | GH¢64,380.00 /  US$11,922.22 |
| 1a | Radio broadcasting | 2 | If you choose this program, you will hear messages on the radio about healthy nutrition for mothers and babies and the importance of attending pre- and post-natal clinics. You will listen to jingles (short slogan) on eating a variety of foods, avoiding sugary drinks, and good hygiene practices. You can listen to dramas (short play) on the harmful effects of alcohol consumption during pregnancy, and benefits of healthy foods resulting in easy and healthy delivery of your baby.  This is a very effective way to send a message to every household for them to practice healthy habits and save lives. | GH¢21,400/  US$ 3,962.96 |
| 1b | SMS and posters | 4 | You will receive SMS through your household head phone about healthy diet choices. Through the SMS, you will learn about vitamins, minerals, and other important nutrients for mothers and babies. You will also be reminded to take your child to the clinic for immunization and for growth monitoring to ensure that your baby is healthy.  You will also see posters at common places in the community showing you that eating healthy food during pregnancy is important to give birth to a healthy baby.  These messages will help you to maintain your child’s health and your own health. | GH¢14,660.00 / US$2,714.81 |
| 1c | Food demonstration in durbars | 5 | There will be meetings organized in your community in the form of a durbar. A professional nurse and nutrition experts will be part of these meetings. There will be live demonstrations of healthy recipes, using simple locally available ingredients. Mothers and pregnant women will learn skills to cook tasty and healthy meals for themselves and their children.  At the durbars you can discuss issues related to good health, required macro & micronutrients, appropriate breast feeding and weaning practices. | GH¢28,320/  US$ 5,244.44 |
| 2 | Youth nutrition education | 8 | Both youth (both in-school and out-of-school children) will be targeted for nutrition education. A nutrition expert will teach them about the link between diet and health, choosing healthy food and the harmful effects of junk food and sugary beverages. Children will also learn about hygiene, healthy lifestyle, and the importance of exclusive breastfeeding. This will encourage children to have a healthy lifestyle from an early age and will grow well and learn better | GH¢49,920/  US$ 9,244.44 |
| 3 | Male involvement in nutrition education | 3 | If you chose this program, men in your community will be organized into groups and given nutrition education. A nutrition expert will advise men about how to take care of pregnant women and children and how to support appropriate feeding and health seeking. The group will empower men to take responsibility for their children’s nutrition and their partners’ well-being.  Lack of male involvement can negatively affect nutrition of children and mothers. When men are more engaged in nutrition, mothers face less hardship and the lives of families can improve. | GH¢17,400/  US$3,222.22 |
| 4 | Livelihood empowerment |  |  | GH¢209,240  US$ 38,748.15 |
| 4a | Water wells and water tanks | 3 | This program will help both men and women in your community to have access to water all year round. Getting water will be less of a daily struggle. This will help you cope with drought, irregular rain and improve poverty. You will be able to maintain vegetable gardens to help improve your nutrition. You will be less vulnerable to hunger and illnesses. | GH¢16,000/  US$ 2,962.96 |
| 4b | Agricultural inputs | 10 | Both men and women in your community will receive agricultural inputs like fertilizers to improve their farming activities. You will be able to maintain or build new farms or vegetable gardens and that will help you get enough of the right foods.  It will also help maintain consistent earning and a healthy family to live a healthy life. | GH¢60,000/  US$11,111.11 |
| 4c | Livelihood skills training | 20 | Both men and women in your community will be enrolled in a community group that improves livelihood. In the group, members will be educated on farming, rearing of animals and other skills such as bread and dress making to improve household earnings.  This program will help you to generate a business idea and will provide funding to help you start the business.  This will support you with additional income to buy a variety of nutritious foods that are needed for your family’s health. | GH¢133,240/  US$ 24,674.07 |
| 5 | Health system strengthening | 2 | During your visit to the health facility or during the health worker’s home visit, you will receive advice on how to improve your nutrition.  You will hear how to have a balanced diet, which is required to keep your baby healthy. You will also learn about healthy weight control and the various nutrients to include in your daily diet to help ensure that you and your baby stay healthy during and after pregnancy. | GH¢12,120/  US$ 2,244.44 |
| 6 | Micronutrient supplementation |  |  | GH¢299,920/  US$55,540.74 |
| 6a | Food fortification | 40 | You will receive specific ready-made nutritious foods that can be added to the existing staple food for children under five years in your household.  This will help the children to be healthy and grow well. | GH¢252,960  US$46,844.44 |
| 6b | Iron-folate supplementation | 7 | Pregnant and breastfeeding women in your communities would receive Iron and Folic acid tablets.  This can prevent them from becoming aneamic during pregnancy and lactating period.  This can prevent learning disabilities, delayed development, and poor physical growth in the baby. It will also ensure that healthy babies are born in the community. | GH¢46,960/  US$ 8,696.30 |
|  | TOTAL | 104 |  | GH¢652,980/  US$120,922.22 |

***NB:*** *A total of 60 stickers were used for the activity*
