# Supplementary material for: What works in engaging communities? Prioritising nutrition interventions in Burkina Faso, Ghana and South Africa
Source: PLoS One. 2023 Dec 13;18(12):e0294410. doi: 10.1371/journal.pone.0294410 (PMC10718458; doi:10.1371/journal.pone.0294410)
Supplement: S3 Appendix — (DOCX) [file pone.0294410.s003.docx]

**Appendix C CHAT participant manual Soweto, South Africa**

**CHAT Manual**

Choosing All Together for Nutrition

in South Africa

Copyright © 2005. The Board of Regents of the University of Michigan.

All Rights Reserved

This version of CHAT was prepared by INPREP Group in collaboration with NIH (funded by the (NIHR) National Institute for Health Research (17\63\154)) using aid received from the United Kingdom in support of Global Health Research.


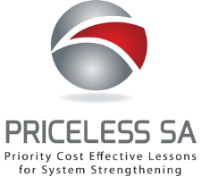


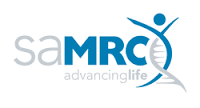

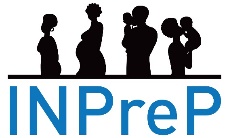

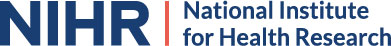


**CHAT**

Choosing All Together for Nutrition

in South Africa

Each page of this manual describes some nutrition services. Choose the nutrition service you want most by placing stickers on the holes on the CHAT board. Use this manual to help you make your choices.

**
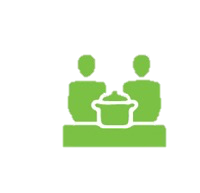
COMMUNITY GARDENS AND CLUBS (1 sticker)**

*By choosing this:*

- There will be a vegetable garden in your community.
- There will be monthly discussion clubs that will help you eat healthier and manage your weight.

**
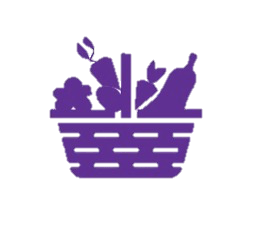
**

**HEALTHY FOOD BASKET (25 stickers)**

*By choosing this:*

- Children will receive a monthly food basket in addition to the Child Support Grant. The basket will have healthy foods.
- Babies and children can eat a healthier diet.
-
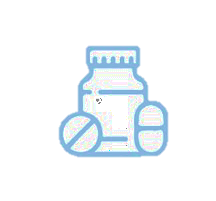
Children will be less hungry and sick.

**PREGNANCY SUPPLEMENTS (1 sticker)**

*By choosing this:*

- Pregnant women will receive 13 extra supplements as well as the regular iron and folate supplements in one tablet. This will help prevent premature delivery and low birth weight.
- Pregnant women and their babies will become healthier.

**
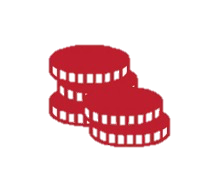
FOOD PRICING (1 sticker)**

*By choosing this:*

- Healthy foods will reduce in price and cost less. Unhealthy foods with too much oil, sugar, and salt will increase in price and cost more.
- You can buy more nutritious food for less money.
- You can improve your diet and keep healthy.

**
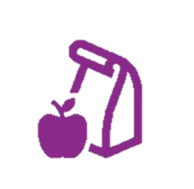
SCHOOL BREEAKFAST (2 stickers)**

*By choosing this:*

- There will be free breakfast for children at schools every day.
  This is in addition to the usual government nutrition programme.
- The extra food will help children learn better and be healthy.


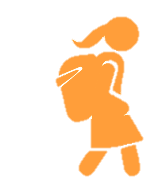
**NUTRITION EDUCATION AND SUPPLEMENTS (2 stickers)**

*By choosing this:*

- Iron and folic acid supplements will be given to teenager girls in schools (because they carry babies). This will prevent girls from getting anaemia (or thin blood).
- **
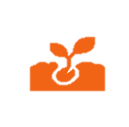
**Children will learn about nutrition *(healthy eating; harms of junk food and sugary drinks; importance of exclusive breastfeeding).*

**SCHOOL FOOD GARDEN (1 sticker)**

*By choosing this:*

- There will be food gardens in schools.
- Children will learn how to grow vegetables. Promote better nutrition.
- The gardens will keep children physically active.

**FOOD SAFETY (2 stickers)**

***
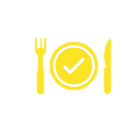
****By choosing this:*

- Street food sellers will learn about food safety and hygiene. The food they prepare will be safe. This will
   prevent illness from food.
- Foods at schools and crèches will be monitored (inspected) to ensure it is fresh and safe to eat. This will protect your children from getting sick from foods.


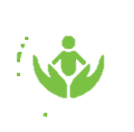
**DAY CARE FOR WORKING PARENTS (11 stickers)**

*By choosing this:*

- There will be low-cost or free day-care for children
- Your child (under 6 years) will be looked after while you are at work.
-
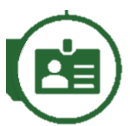
You do not have to miss work.

**LINK TO JOBS (1 stickers)**

*By choosing this:*

- You will receive help to apply for jobs.
- You don’t have to look for jobs alone, a social worker will help.
- You might learn about new opportunities.

**
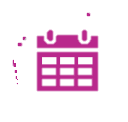
PAID MATERNITY LEAVE (8 stickers)**

*By choosing this:*

- Moms will receive income during maternity leave up to 6 months (formal and informal sector).
- This helps lessen the worry of money.
- Moms can stay home longer with their babies. They can breastfeed longer.

**
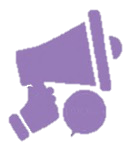
NUTRITION COMMUNICATION (2 stickers)**

*By choosing this:*

- You will see information in the media about how to feed your baby (breastfeeding, complementary feeding).
- Posters, films, and radio messages will encourage men to be involved in caring for the baby.
- Moms will receive regular SMSs (on healthy diet, vitamins, important foods for moms and babies) and clinic reminders.


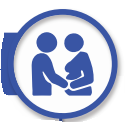
**COUPLE ANTENATAL EDUCATION (7 stickers)**

*By choosing this:*

- Mothers and their partner will receive education at antenatal clinics. They will learn about:
  - nutrition during pregnancy
  - how to care for a pregnant woman and a baby
  - how to feed babies appropriately.
- Men will learn how to support pregnant women and mothers.

**
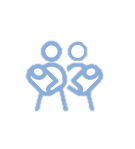
**

**MOTHERS NUTRITION SUPPORT (7 stickers)**

*By choosing this:*

- Moms will get individual nutrition advice at the clinic (from breastfeeding specialists).
  - Learn about healthy diets and healthy weight.
  - Why moms should avoid ash or soil, ice, alcohol, tobacco, and drugs.
- New moms will be visited 3 times at home by a community health worker.
  - Examine the baby and the mom.
  - Screen the mom for depression.
  - Help moms with any questions about nutrition.
- Monthly meetings for pregnant women and moms at the clinic:
  - A lady doctor, nutritionist and community health worker will attend.
  - Live demonstrations of healthy recipes.
