# Supplementary material for: What works in engaging communities? Prioritising nutrition interventions in Burkina Faso, Ghana and South Africa
Source: PLoS One. 2023 Dec 13;18(12):e0294410. doi: 10.1371/journal.pone.0294410 (PMC10718458; doi:10.1371/journal.pone.0294410)
Supplement: S5 Appendix — (DOCX) [file pone.0294410.s005.docx]

**Appendix E CHAT Evaluation coding framework**

Research Question:

1. How well were participants able to complete the activities of the CHAT process?

| Code and sub-code name | Code description | Burkina Faso quotes | Ghana quotes | South Africa quotes |
| --- | --- | --- | --- | --- |
| 1. How well were participants able to complete the activities of the CHAT process? |  |  |  |  |
| 1. Deliberating trade-offs | Participants considered which interventions to prioritise with the allocated stickers. | Interviewer: Ok, those who didn’t choose it, why didn’t you choose number 7?  Participant: It’s because we didn’t have enough money otherwise, we would have bought it all.  Participant: Same over here, it’s because we didn’t have enough money.  Participant: Us too, it’s because there wasn’t enough money otherwise it’s good. FGD07_Women_35-55yrs_Nanoro, Burkina Faso  We didn’t choose it, but not because it is not a good programme. It’s like if you went to buy things at the market, and you didn’t have enough money, so we have paid for what was important to us, and we didn’t have enough money, otherwise we would have chosen it. FGD10_Women_18-34yrs_Nanoro, Burkina Faso  Facilitator: Because, Madame, you spoke about money, you’re also saying that it was the money that wasn’t enough. Are you sure?  [Laughs]  Participant: Yes.  Facilitator: And you, Madame?  Participant: We didn’t have enough money.  [Laughs]  Facilitator: It was also a lack of money?  Participant: … Yes, yes.  FGD11_Women_26-34yrs_Nanoro, Burkina Faso | What I understand is that the interventions we have seen are those we are going to buy with our money with the stickers given to us and those that we choose are those we need most in our families or community.  FGD05_Men_24-34yrs_Navrongo, Ghana  It is good but just that our money was not enough for us to buy everything. FGD03_Women_26-39yrs_Navrongo, Ghana  My partner and I chose youth nutrition, education, agricultural inputs, livelihood skills training, water tanks, food fortification, health system strengthening and the rest of our money went to iron folic supplementation.  FGD06_Women_40-50yrs_Navrongo, Ghana | I was referring to it as awkward because when we checked our prices and other things, we had to make changes so that it can achieve what we wanted.  FGD18_Men_18+_Soweto, South Africa  I also feel uhm... about prioritise like he said earlier on it was more about prioritise than the individual so what I will normally prioritise sometimes might not come that important for the other person. FGD21_Mixed_18+_Soweto, South Africa |
| 2. Agreement on priorities | The participants came to a consensus on which interventions to prioritise. | We are satisfied with our group choice.  FGD10_Men_18-34_Nanoro, Burkina Faso  Facilitator: Is there anyone who would like to make a change here?  Participant: No, the board is perfect. It’s what we want.  Participant: Unless you were going to give us more money to buy the programme. [All Laugh] FGD07_Women_35-55yrs_Nanoro, Burkina Faso  We are happy with our choice; now we count on your support to implement these projects in our village. FGD08_Women_18-25yrs_Nanoro, Burkina Faso | For my side I think there are no modifications because those are what we want and we also selected them based on our needs in community.  FGD05_Men_24-34yrs_Navrongo, Ghana  I think if we get the agricultural inputs and the other nutrition interventions we can now sit together and plan together so that we will have a common consensus on which will improve our well-being and also work hard to improve nutrition decisions in Biu. FGD06_Women_40-50yrs_Navrongo, Ghana  What I want to say is that, even as you are staying with others, your child for instance might have a different opinion about something, so what we chose, we are confident that it would bring good results. We are only pleading that it won’t bring about something bad, the thing is that, because you cannot know what someone is thinking. FGD02_Men_35-50yrs_Navrongo, Ghana | *We had come to an agreement and this thing was resolved and we thought of how we could change it* FGD18_Men_18+_Soweto, South Africa  Facilitator: Okay. And is there anyone who would like to comment on participant 5's choice? Who would like to support it or not support it?  Participant: We support it. She is correct. Sometimes, the child comes from a home which has nothing...Homes are not similar...You find that my child will leave without eating anything and with nothing for lunch as well. When the child arrives at school, they would be able to receive porridge or soup of whatever food which would help the child to be alright. FGD17_Women_50+yrs_Soweto, South Africa  Facilitator: Does anybody agree or disagree with that? P1 what do you think?  Participant: Uhm. I agree with her. Because uhm... This thing starts at home and when it starts at home it is easy to confront someone else in saying no, for this thing to be correct it must say this and that so if eh... You doing things alone by yourself and do not care about the community then I do not think it can work but if you do it knowing that what I am doing it will help the community and that if you are the man and female you must be taught how to respect another member of the community and then if you are a person like that it is easy for a person to come to you and ask for help but if you have this thing of being rude when people come to you for help but a person who is able to say I know how we can solve something like this you see and it becomes easy for the community to come to them and say we are asking for help in this, because we saw you do something like this we just wanted to know how you did it, someone’s knowledge can be able to help the community.  FGD20_Women_18-49+yrs_Soweto, South Africa |
| 3. Working together | The participants spoke of how they worked together throughout the CHAT activity. | If we work together and there is help, I know that there will be change.  FGD09_Women_35-55yrs_Nanoro, Burkina Faso  Facilitator: How did you find working in groups of two?  Participant: It was good and made us discover things. FGD12_Women_26-34yrs_Nanoro, Burkina Faso  We think that this was good because nobody forced anyone else to choose; each person was able to choose what she wanted and could tell us the reason that drove her to make those choices. FGD08_Women_18-25yrs_Nanoro, Burkina Faso | Coming together gives sound mind to everybody when you are in the field of work. Working together will let us get what we need without any misunderstanding. I think as we all come together as one and make our choices we will still continue to work hard to achieve our aim as a team.  FGD05_Men_24-34yrs_Navrongo, Ghana  Facilitator: You that have all come together, how do you think coming together, how difficult is that?  Participant: Coming together to work is very interesting to us. All of us are here in Manyoro but we don’t meet each other to exchange greetings, but as we have come together here, some people said things that are pleasant to us, if it is that all of us come together like that to work, we know we can. FGD01_Women_40-50yrs_Navrongo, Ghana  As we are gathered here, we have to accept each other’s view. FGD02_Men_35-50yrs_Navrongo, Ghana | I think the group setting helped us a lot because we come from different places and backgrounds so we can elaborate things to you, and it also shows you the other side of the coin which you might not know so it helps a lot because we learn from each other.  FGD19_Men_50+_Soweto, South Africa  As an individual there were a lot of things that I knew but there's a lot of things that I'd forgotten as well that I got reminded of today, especially being here with the team. The things that I did know that the team was not aware of, I'm glad I could be a part of them learning and a part of me also being able to share with them the information that I knew. I think as a team we come from quite different places and different communities. FGD22_Mixed_18+yrs_Soweto, South Africa  Personally, my take home for today, we do not all come from the same and we must appreciate what ever that sort of thing you have or get at home, whether you get it from home or community, but it goes back to appreciate what you have because somebody else might have at that current moment. It taught me to be a better listener and see where I can help my community and how to prevent it. FGD21_Mixed_18+yrs_Soweto, South Africa |
| 4. Having a shared vision | The participants recognised the common ways that the community need to bring about change. This included making future plans to support their community to work together. | As far as we are concerned I don’t think that anything could hinder the implementation. Our whole community needs this help. Nobody could say that they did not need this help.  FGD08_Women_18-25yrs_Nanoro, Burkina Faso  As it is an aid that is the problem, we can't refuse them, otherwise we would have liked to choose. We pray to God that what you choose works, that’s what we want. FGD10_Men_18-34yrs_Nanoro, Burkina Faso  As far as we, the men, are concerned, we can put little groups in place, and we will choose people to be responsible for organising the work. Everyone will be able to have their field, however, we can identify 2 hectares of space for the youngsters; in this space we can have a variety of crops. We can sew corn, sesame, artichokes. We are going to let the community leaders accompany them.  FGD13_Women_26-34yrs_Nanoro, Burkina Faso | What I think is that if everybody has his/her hand work to do and nobody is looking up to another person for support each day and night, It will help us to bring change in our lives because that will help your wife, the children and yourself to get nutritious food to eat every day.  FGD04_Men_35-50yrs_Navrongo, Ghana  For us, we want to take care of our children very well but the problem is that we don’t have money and when we wake up. FGD06_Women_40-50yrs_Navrongo, Ghana  When we leave here it does not mean everything ends here. we will still have to meet as a group and discuss how we will be able to eat nutritious foods and it will also serve as opportunity for people to share with us how they get their healthy foods but if we leave here and everything ends here then I do not think we can improve nutrition in this community. FGD03_Women_26-39yrs_Navrongo, Ghana | I think it was easy I will just go back to the point that I made, it becomes easier if you see this kind of things in our everyday life and not every one of them is easy but some of those decisions were not that hard to make because it is what we see daily.  FGD21_Mixed_18+_Soweto, South Africa  The exercise is clear, but I think maybe the outcome of this can be implemented to make these women's lives better. FGD19_Men_50+yrs_Soweto, South Africa  I think uhm... we must, together with the community, do the community garden, so that we can be able to help other people who are not working.  FGD20_Women_18-49_Soweto, South Africa |
| 5. Increasing knowledge | The knowledge gained from the CHAT activity and the ways this knowledge would be shared with community members who were not participating. | I’m an old lady so it was difficult. [Laughs] When we got the cards, I didn’t understand a thing. I just wanted to take the stickers and line them all up and despite that, we paid, and that’s it. [Laughs] But the explanations I could understand. If I go back, I’m going to say to my daughters-in-law to come and I will teach them.  FGD09_Women_35-55yrs_Nanoro, Burkina Faso  What we have chosen, was part of our daily life. There were some things that we didn’t know and we have learnt them here today. FGD15_Leaders_18+yrs_Nanoro, Burkina Faso  What we can do is return home, see our families and teach them what we have learned here. FGD14_Women_18-24yrs_Nanoro, Burkina Faso | What I have to say is that, what you people taught us here; when we go home we should also teach our colleagues and we come together as one team to work, to improve nutrition for the community.  FGD05_Men_24-34yrs_Navrongo, Ghana  I think when we go home, we can tell others what we have learnt here. FGD01_Women_40-50yrs_Navrongo, Ghana  To me, I think this exercise is something that has helped us, most of us don’t know what nutritious foods are but we have learned a lot concerning nutrition from this exercise. FGD04_Men_35-50yrs_Navrongo, Ghana | It was difficult, but we learnt something, and we will be able to help others with problem solving.  FGD22_Mixed_18+_Soweto, South Africa  As participant 2, when I reflect, this has helped me immensely because it was the first time for some of us or a few of others, to communicate how you view things. Sometimes you would read about something and you do not also understand how things should proceed, but through engaging in these discussions, I have learnt much more than when I read about those things. It also provided me with a platform to also ask other people. FGD17_Women_50+yrs_Soweto, South Africa  Participant: We learnt immensely today - things we were not aware of. Issues relating to mothers, children and breastfeeding. I now know that breasts can  Facilitator: The nipple can crack.  Participant: Yes. I didn't even know that. Thank you. FGD18_Men_18+yrs_Soweto, South Africa |
| 6. Having a skilled facilitator | Facilitators checking in with the community members to see if they understood the CHAT, using Healthy Conversation Skills and attempting to include different participants to increase inclusivity of the CHAT activity. | Facilitator: Ok. But how are you going to do that, to choose the project which will be the most beneficial for you?  FGD07_Women_35-55yrs_Nanoro, Burkina Faso  Facilitator: Ok, who wants to add to what this lady just said? FGD08_Women_18-25yrs_Nanoro, Burkina Faso  Facilitator: Have you all understood the eight programmes?  Participant: Yes, yes.  Facilitator: Okay. In this case my neighbours on the left are going to explain to me the first programme. The person who would like to add something just puts up their hand. If you haven't understood, we can go over it again so that you can understand it better.  Participant: It's a woman who has a tool; another woman who is carrying a basket on her head; and another who is cooking. FGD11_Women_26-34yrs_Nanoro, Burkina Faso | Facilitator: What is the aim of this activity?  Participant: The reason why we are here is that, what we can do to be able to take care of our families and what we can do to get something to eat. Those are the reasons why we are here to discuss and get solutions for healthy life in our jurisdiction. This will make us grow strong, healthy and have the strength to do whatever we want.  FGD04_Men_35-50yrs_Navrongo, Ghana  Facilitator: Who has something different to say, who doesn’t agree to what they are saying? FGD01_Women_40-50yrs_Navrongo, Ghana  Facilitator: Yes, I agree, but what was it that I just said to do as an activity?  Participant: What we have to do is choose the projects which are going to help us have good health. FGD07_Women_35-55yrs_Navrongo, Ghana | Facilitator: Does anyone want to add anything?  FGD19_Men_50+_Soweto, South Africa  Facilitator: Does anyone else have something to add on to this scenario? FGD21_Mixed_18+yrs_Soweto, South Africa  Facilitator: Okay...Did ma read the description of the healthy food basket so that you know what entails?  Participant: Ma?  Facilitator: Have you read it, ma?  Participant: Yes, we have read it. FGD17_Women_50+_Soweto, South Africa |
| 7. Sharing power | The power balance between research team and participants. | Yes, it can help. The fact that you have taken us here and enlightened us, we can follow it.  FGD09_Women_35-55_Nanoro, Burkina Faso  Interviewer: Who else would like to say something? Gurunga has said what she thinks. My neighbour, I’m listening to you.  Participant: Everything was interesting. What we’d like now is support. FGD11_Women_26-34yrs_Nanoro, Burkina Faso  Speak up please. While the child is crying I cannot hear well. FGD12_Women_26-34yrs_Nanoro, Burkina Faso | The reason why we say it can be done is, you are bringing help, you are going to help us, so who is there and the person doesn’t want good health, if you are not healthy you cannot do anything, so we know by the help of God it will work. FGD01_Women_40-50yrs_Navrongo, Ghana  I think if we want it to work unless the Navrongo Research Centre helps us because we are like blind people and you people came to open our eyes. We think today will not be your last coming to this community. If you people continue coming to educate us, we will then learn how to improve nutrition in Janania and start our own nutritional programmes for development of the community. FGD05_Men_24-34yrs_Navrongo, Ghana  After listening to what you people read, I know that most of our interventions or programmes with help us a lot especially the livelihood skills training, male involvement in nutrition education and agricultural inputs. FGD06_Women_40-50yrs_Navrongo, Ghana | I was grateful to be a part of this team. I learnt quite a bit of things today and just want to thank you guys for giving us this opportunity to be able to come and meet and be a part of this discussion. My wish is that you guys would be able to go out into the community to be able to teach more people out there in the same way that you guys have taught us.  FGD22_Mixed_18+_Soweto, South Africa  I... I am also grateful to people that are like me...that like to speak because we like to speak so that we do not bottle things up. But, we cannot as there is no 1 who asks us, what do you want and what is happening. Things that bother us are always taken as a "political" issue but they do not come to us and sit with us, unless there is an issue to be resolved - unlike, how we are sitting today and discussing what is happening around us. They do not speak to us. FGD17_Women_50+yrs_Soweto, South Africa  I will give you the chance then we will move on to the next card ladies because we have spent a lot of time on this. FGD20_Women_18-49yrs_Soweto, South Africa |
