# Supplementary material for: What works in engaging communities? Prioritising nutrition interventions in Burkina Faso, Ghana and South Africa
Source: PLoS One. 2023 Dec 13;18(12):e0294410. doi: 10.1371/journal.pone.0294410 (PMC10718458; doi:10.1371/journal.pone.0294410)
Supplement: Attachment — Submitted filename: CHAT process evaluation_PLOSONE_response to reviewers v2.docx [file pone.0294410.s006.docx]

**Dr. Stephen Apanga**

PLOS ONE

23rd October 2023

Dear Dr Apanga,

**Manuscript PLOS ONE. ‘What works in engaging communities? Prioritising nutrition interventions in Burkina Faso, Ghana and South Africa’ - PONE-D-23-18772**

We thank the reviewers for their helpful comments on our manuscript and to the editors for their consideration.

We also confirm that “The funders had no role in study design, data collection and analysis, decision to publish, or preparation of the manuscript”.

Below, we respond to the points raised by reviewers and have attached both a revised clean version of our manuscript and one with tracked changes as requested. No more references have been added but the reference list order has been updated.

Best wishes,

Dr Daniella Watson

Postdoctoral Research Associate & Health Psychologist

King’s College London, UK

**Manuscript PLOS ONE. ‘What works in engaging communities? Prioritising nutrition interventions in Burkina Faso, Ghana and South Africa’ - PONE-D-23-18772**

|  | **Comment** | **Author Response** | **Page No. of tracked-changes Manuscript** | **Text Excerpt** |
| --- | --- | --- | --- | --- |
| **Reviewer 1** | | | | |
| 1 | In this manuscript authors conducted a process evaluation of using the CHAT tool to encourage community engagement and participation in the prioritizing of nutrition interventions. The manuscript is well written and makes a contribution to what works in involving community members in prioritizing nutrition interventions to encourage a sense of ownership and sustainability of such interventions. However, I have a few issues I will like the authors to address. | We thank the reviewer for their thoughtful feedback. We have incorporated the reviewer’s comments and believe that they have improved this paper. |  |  |
| 2 | In the introduction section, line 120, authors have indicated that this is the first time the CHAT tool is used in the study setting. Although, this is agreeable, there is a published paper (Link here: <https://pubmed.ncbi.nlm.nih.gov/36962493/>) using CHAT tool in Navrongo, Ghana authored by some of the authors in the current manuscript. Although the work is cited in the manuscript, I will suggest it is cited earlier preferably in the background in which authors would explain the added value the current manuscript is bringing that is different from the already published work. What questions were unanswered in the published work from Navrongo, Ghana which are now being answered in the current manuscript? These should be well articulated in the background section. | We thank the reviewer for their comment and have introduced the three CHAT studies in the Introduction. This includes findings on the community nutrition priorities in Burkina Faso, Ghana and South Africa and what this paper adds to their findings in terms of the process evaluation of the CHAT tool. | Page 6  Lines 130-142 | At the time of the study described in this paper, CHAT had not been implemented with community members in Burkina Faso, Ghana, or urban South Africa, nor been used to prioritise nutrition interventions; the way CHAT works in these contexts and with this topic was not understood. Our research team, the Improved Nutrition Preconception, Pregnancy and Post-delivery (INPreP)^(14)^, were the first to adapt CHAT for those specific populations and for nutrition interventions. INPreP aimed to identify feasible and appropriate maternal and infant nutrition interventions through community engagement in Burkina Faso, Ghana and South Africa^(14)^, implementing CHAT to understand the community members’ programme priorities for improved nutrition. In Burkina Faso, the community members prioritised women’s capacity through agriculture, mass communication about nutrition, and child-friendly school initiatives^(15)^. In Ghana, the community members prioritised livelihood empowerment, male involvement and micronutrient supplementation^(16)^. In South Africa, the community members prioritised school breakfasts, six-months paid maternity leave, and improved food safety in South Africa^(17)^. This paper adds to the community priorities from the above publications and addresses the gap in evaluation of community engagement approaches such as CHAT, which Butterfoss (2006) highlighted as important to understand how community engagement works^(12)^. |
| 3 | In line 220 of the methods section, check for spelling of HIV/AIDS | Thank you, we have edited the word. | Page 10  Line 249 | Health challenges in Soweto include high rates of HIV/AIDS infection, tuberculosis, rising rates of diabetes, infant and maternal mortality, road traffic incidents, intimate partner crime, rape and murder. |
| 4 | In the methods section line 285, the site for selecting the participants in Soweto has the tendency of selection bias which may not be representative of the entire community. Authors could justify their choice of such an approach given that persons who do not seek for public transport from the hospital's taxi rank are obviously excluded. The approach is obviously different from the approach adopted in Navrongo and in Burkina Faso. Or they could cited it as a limitation of the study. | We have taken the reviewer’s latter suggestion to cite the selection bias in the strength and limitation sub-heading of the discussion. Thank you for the suggestion. | Page 24-25  Lines 567-576 | **Strengths and limitations**  The evaluation was constrained by COVID-19 pandemic restrictions. During this time, we had both travel restrictions and extra COVID-19 work to balance with conducting CHAT. Interviews with and in-person observations of CHAT participants and facilitators to explore their experiences might have given a more in-depth understanding of whether CHAT was acceptable and how it works. Yet our teams did not have the capacity to conduct these methods at the time.  In Soweto participants were recruited using an established recruitment strategy which has been used in previous research and has been effective in capturing the diversity of sociodemographic characteristics of Soweto's inhabitants^(45, 50)^ .  The approach involved approaching prospective participants at a hospital taxi rank and it is possible that the entire community was not represented using this method. Despite these challenges, a substantial qualitative analysis of 22 CHAT transcripts was conducted. The wider INPreP team supported the analysis which elicited a wider interpretation and increased cultural relevance. |
| **Reviewer 2** | | | | |
| 5 | This is a well written paper which is a huge contribution to the limited literature in the field of deliberative pooling and especially in finding solutions to the challenges of maternal and child nutrition services in sub Saharan Africa. | We appreciate the reviewer’s positive review of this manuscript. Thank you. |  |  |
| 6 | Methods and materials The word “identity” on line 130 should be “identify” | Thank you, we have edited the word. | Page 6-7  Lines 150-152 | We used the UK MRC guidance on process evaluation of complex interventions to identify the mechanisms of impact of CHAT and to consider how context and implementation influenced the CHAT process^(15)^ |
